# A Minimum of Three Motifs Is Essential for Optimal Binding of Pseudomurein Cell Wall-Binding Domain of *Methanothermobacter thermautotrophicus*

**DOI:** 10.1371/journal.pone.0021582

**Published:** 2011-06-27

**Authors:** Ganesh Ram R. Visweswaran, Bauke W. Dijkstra, Jan Kok

**Affiliations:** 1 Department of Molecular Genetics, Groningen Biomolecular Sciences and Biotechnology Institute, University of Groningen, Groningen, The Netherlands; 2 Laboratory of Biophysical Chemistry, Groningen Biomolecular Sciences and Biotechnology Institute, University of Groningen, Groningen, The Netherlands; Semmelweis University, Hungary

## Abstract

We have biochemically and functionally characterized the pseudomurein cell wall-binding (PMB) domain that is present at the C-terminus of the Surface (S)-layer protein MTH719 from *Methanothermobacter thermautotrophicus*. Chemical denaturation of the protein with guanidinium hydrochloride occurred at 3.8 M. A PMB-GFP fusion protein not only binds to intact pseudomurein of methanogenic archaea, but also to spheroplasts of lysozyme-treated bacterial cells. This binding is pH dependent. At least two of the three motifs that are present in the domain are necessary for binding. Limited proteolysis revealed a possible cleavage site in the spacing sequence between motifs 1 and 2 of the PMB domain, indicating that the motif region itself is protected from proteases.

## Introduction

The major component of bacterial cell walls is peptidoglycan, also called murein. Several proteins bind to peptidoglycan *via* a highly conserved Lysin Motif (LysM) domain, which allows their non-covalent attachment to bacterial cells [4], [14]. LysM domain-containing proteins have not only been found in bacteria, but they also occur in lower and higher eukaryotes, ranging from yeast to plants and animals [4].

In contrast, methanogenic archaea have a layer of pseudomurein, which differs from murein in that the *N*-acetylmuramic acid (NAM) residues, present in murein, are substituted by *N*-acetyltalosaminuronic acid (NAT) residues [7], and in that the ß-1,4-glycosidic linkages of murein are replaced by ß-1,3-glycosidic bonds between *N*-acetyl-D-glucosamine (NAG) and NAT. As a consequence, most of the enzymes that hydrolyze the glycosidic bonds of the bacterial cell wall, including lysozyme, are ineffective in degrading the pseudomurein-containing archaeal cell wall [12].

Enzymes that are specific for the degradation of pseudomurein have been identified in archaeal-specific prophages. Because they cleave the Ala-є-Lys isopeptide bond in the peptide linkers that connect adjacent pseudomurein layers, they have been named pseudomurein endoisopeptidases (Pei) [9], [16]. So far, two such peptidases have been described, PeiW (UniProtKB Q7LYX0, 284 amino acid residues) and PeiP (UniProtKB Q77WJ4, 305 amino acid residues) [2], [5], [8], [10]–[11], [16] with apparent molecular masses of 33 [8], [9] and 36 kDa, respectively [Visweswaran et al., unpublished results]. They have a similar modular architecture, with the cell wall lysing activity located in a C-terminal cysteine protease domain. The N-terminal part of the two enzymes contains four so-called pseudomurein binding motifs, together forming a pseudomurein cell wall binding domain, or PMB domain [16]. The PMB domain helps in binding of the Pei-enzymes to the substrate and facilitates cleavage of the isopeptide bond [9], [16]. One PMB motif sequence contains about 30–35 amino acids with highly conserved hydrophobic residues such as proline, isoleucine, leucine, valine and phenylalanine (HMM logo, http://pfam.sanger.ac.uk/family?acc=PF09373#tabview=tab3).

PMB domains are similar to LysM-domains with respect to their variable location in proteins; they can be present at the N- as well as the C-terminus or even in the middle of a protein sequence ([4], http://www.ebi.ac.uk/interpro/ISpy?ipr=IPR018975&tax=28890). Moreover, both domain types have variable iso-electric points, ranging from 3–10 [4]. Major differences include the evolutionary conservation, the lengths of the motifs, and their binding specificities. The PMB domain contains 1 to 4 motifs of around 30–35 amino acids and specifically binds to pseudomurein, while the LysM domains contain 1 to 6 motifs of 44–65 amino acids and bind to murein. The LysM domain recognizes the *N*-acetyl-D-glucosamine (NAG) moiety of murein [3], [4], a sugar moiety that is also present in pseudomurein. In contrast to the LysM domain, the PMB domain is not widely distributed; so far it has been found in two archaea (*Methanothermobacter thermautotrophicus* (MTH) and *Methanosphaera stadtmanae*), in two archaea-specific viruses (*Methanobacterium* prophage and *Methanothermobacter* prophage), and in a few species of bacteria (*Xanthomonas campestris*, *Granulibacter bethesdensis* and *Novosphingobium aromaticivorans*) [16]. Interestingly, these three bacteria contain LysM proteins as well as proteins carrying one or more PMB motifs (data not shown).

**Figure 1:**
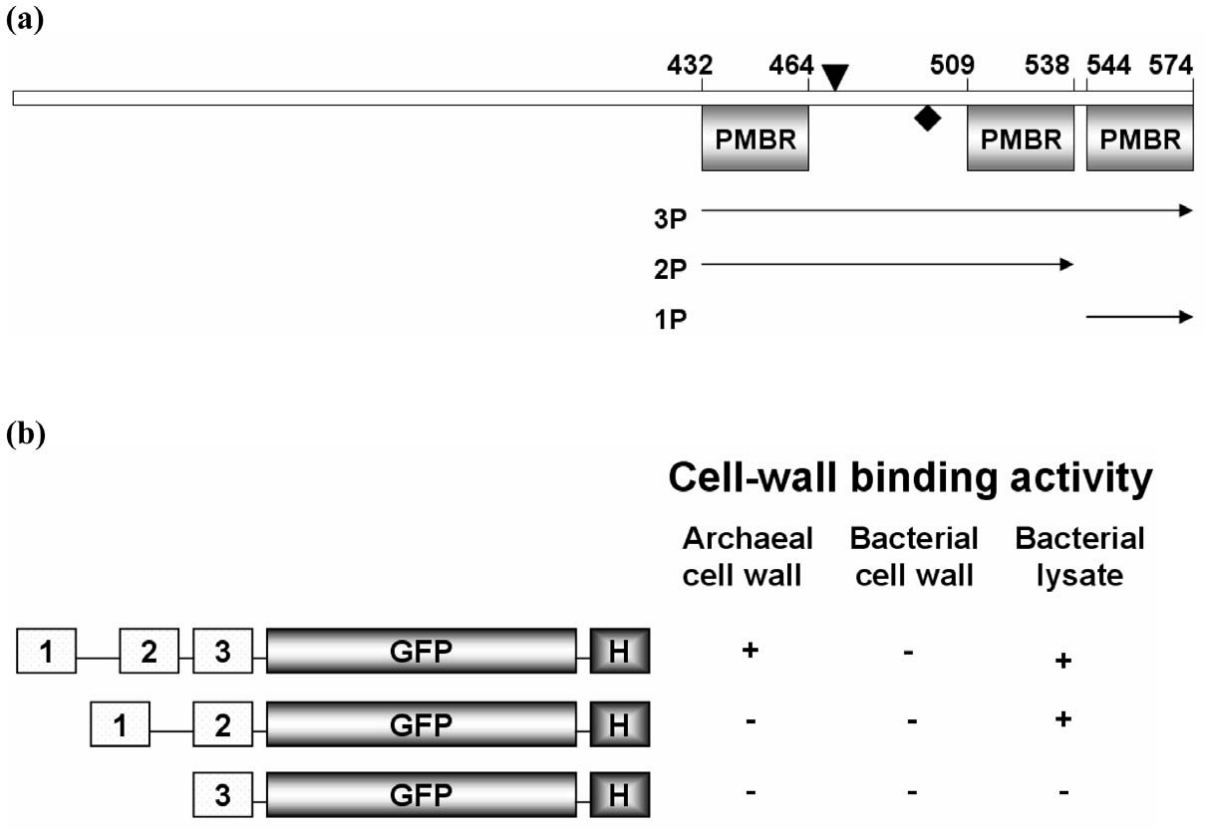
Construction and binding of MTH719 PMB-GFP fusion variants. (**a**) Schematic view of the 574-residue S-layer protein MTH719 with predicted PMB domains. (▾) and (♦) represent the major trypsin and chymotrypsin cleavage sites, respectively, as predicted by Peptide cutter (ExPASy). Arrows labeled 1P, 2P and 3P show the regions used for the GFP fusion proteins. (**b**) Molecular architecture of different PMB fusion constructs. Numbers indicate the different PMB motifs (see Fig. 1a). GFP and H represent Green Fluorescent Protein and the Histidine_10_ tag, respectively. At the right, cell wall binding activity is denoted qualitatively.

We are interested in the biochemical and molecular functions of the PMB domain, which is rather poorly studied when compared to its eubacterial counterpart, the LysM domain. Therefore, the PMB domain of MTH719 (UniProtKB O26815), a protein that has been annotated as one of the putative Surface (S)-layer proteins in the Gram-positive methanogen *M. thermautotrophicus* has been studied. MTH719 is composed of 574 amino acid residues and carries a C-terminal PMB domain-containing three motifs (Fig. 1a) and an N-terminal signal sequence of 27 amino acid residues (BioInfo Bank, http://rpsp.bioinfo.pl/). Blast searches with the protein sequence of MTH719 showed that it is homologous to various other S-layer proteins, supporting the labeling of MTH719 as a possible S-layer protein. Our functional studies with motif deletion constructs revealed that the PMB domain of MTH719 not only binds to pseudomurein cell wall-containing archaea but also to cell wall fragments on bacterial spheroplasts. For this binding, at least two motifs are required and it is pH dependent. Biochemical studies on the three-motif domain showed that it is stable and forms multimers in solution at pH 7.0.

## Materials and Methods

### Strains and plasmids

*Escherichia coli* was grown with shaking at 37°C in LB broth (Difco, Sparks, MD, USA), containing ampicillin (50 µg/ml) or chloroamphinicol (50 µg/ml), as required. *Lactococcus lactis* NZ9000 was grown in M17 broth (Difco) containing 0.5% glucose (GM17) at 30°C as standing cultures. Gene constructs used in this study were made in pBADcLIC and pBADcLIC-GFP (Green Fluorescent Protein), bearing an arabinose inducible promoter, using the Ligation Independent Cloning (LIC) strategy described previously [1]. Primers used for the amplification of specific DNA fragments are shown in Table S1. Ligation mixtures of plasmids and PMB DNA fragments were used for transformation of *E. coli* Rosetta gami 2 (Novagen, Darmstadt, Germany) using the heat shock method [15]. Cells were plated on selective LB 1.5% (w/v) agar plates. Transformants were checked by colony PCR and plasmid DNA sequencing (ServiceXS, Leiden, The Netherlands). The fusion of the PMB domain of PeiW to GFP-His_6_ was made previously [2].

### Protein expression and purification

C-terminally His-tagged and GFP-His_10_ fusion proteins were expressed and purified according to the Qiagen Ni-NTA protocol. Purified protein was dialyzed overnight for buffer exchange in a 3.5-kDa molecular weight cut off (MWCO) dialysis bag (Spectra/por, Spectrum laboratories, Inc., Rancho Dominguez, CA, USA). Dialyzed protein was concentrated using a protein concentrator device with a 5-kDa MWCO filter (Millipore, Billerica, MA, USA).

### Native PAGE, in-gel fluorescence and Western hybridization

Ni-NTA-purified and concentrated protein was loaded on a non-denaturing 15% polyacrylamide gel. Electrophoresis was performed at 30 mA/250 V under neutral pH conditions. Gels were stained with colloidal Coomassie brilliant blue. All GFP fusion protein samples were subjected to SDS 10%-PAGE gels and in-gel GFP fluorescence was visualized using a Gel Documentation System (Bio-Rad Laboratories Inc, Hercules, CA, USA). Gels were blot-transferred onto polyvinylidene fluoride transfer membranes (GE Healthcare UK Limited, Little Chalfont, Buckinghamshire, UK). A SNAP-id system (Millipore) was used for washing and incubation steps according to the protocol of the manufacturer. Anti-His-tag polyclonal rabbit IgG (Santa Cruz Biotechnology, Santa Cruz, CA, USA) and anti-rabbit IgG peroxidase (GE Healthcare UK Limited) were used as primary and secondary antibodies, respectively. Enhanced chemiluminescence was used to detect the signals, according to the supplier's protocol (GE Healthcare UK Limited).

### Tryptophan fluorescence

Purified three pseudomurein binding motifs with C-terminal ten-Histidine tag (3P-His_10_) at 3 µM end concentration was incubated in increasing concentrations of 0.2 to 5.0 M guanidinium hydrochloride in 50 mM NaH_2_PO_4_, pH 8.5, for at least 1 h at 25°C, after which fluorescence intensity was measured using a Luminescence spectrometer (AMINCO Bowman series 2, Madison, WI, USA) in a cuvette with a path length of 1 cm. The excitation wavelength was set to 295 nm and the emission spectra were recorded between 300 to 450 nm. The respective blanks were subtracted from the spectra and normalized data were used. All spectra were accumulated after two successive scans.

### Analytical chromatography

Size exclusion column chromatography (Superdex 200 10/300 GL; GE Healthcare, Uppsala, Sweden) was used to investigate the apparent molecular mass of 3P-His_10_, protein. Initially the column was calibrated with globular proteins of known molecular mass like thyroglobulin (669 kDa), ferritin (440 kDa), aldolase (158 kDa), conalbumin (75 kDa) and ovalbumin (43 kDa). Blue dextran was used to determine the void volume (V_o_) of the column. Chromatography was performed at room temperature in 50 mM NaH_2_PO_4_, 150 mM NaCl, pH 7.0 and pH 9.0. The partition coefficient (K_av_) was determined by the formula K_av_ = (V_e_-V_o_)/(V_t_-V_o_), where V_e_ is the elution volume, V_o_ the void volume and V_t_ the total volume of the column. The apparent molecular mass was determined by the inverse logarithm of the partition coefficient.

### Partial proteolysis

Proteolytic degradation of purified 3P-His_10_ with chymotrypsin and trypsin (both at 4.5 and 45 µg/ml) was performed for 30 min at 25°C in 50 mM NaH_2_PO_4_, pH 8.5. The reaction was stopped by adding 6 mM (end concentration) of phenyl methyl sulphonyl fluoride (PMSF) (Merck KGaA) followed by boiling for 10 min on a heating block. Digestion products were examined by SDS-20% PAGE with undigested protein as the control. Major cleavage sites for chymotrypsin and trypsin were predicted by the Peptide Cutter program (ExPASy, http://www.expasy.org/tools/).

### Fluorescence microscopy

*L*. *lactis* spheroplasts were obtained by incubating a 100-fold concentrated overnight culture in GM17 with lysozyme (10 mg/ml) at 56°C for 1 h followed by three washing steps with 50 mM NaH_2_PO_4_, pH 8.5. Proper spheroplasting was confirmed by a rapid spheroplasts lysis test using 0.5% SDS (Fig. S1). Subsequently, the spheroplasts were spun down at 20800× g in an eppendorf centrifuge; the pellet was washed thrice with 50 mM NaH_2_PO_4_, pH 8.5 and finally resuspended in the same buffer. Three pseudomurein binding motifs with C-terminal GFP and ten-Histidine tag (3P-GFP-His_10_), two pseudomurein binding motifs with C-terminal GFP and ten-Histidine tag (2P-GFP-His_10_) and one pseudomurein binding motif with C-terminal GFP and ten-Histidine tag (1P-GFP-His_10_) were each incubated with a mixture of pseudomurein of cells of *Methanobacterium* sp. (Sigma-Aldrich, Zwijndercht, The Netherlands) and *L. lactis* spheroplasts at room temperature for 30 min. Simultaneously, the PMB domain of PeiW-GFP-His_6_ was incubated with *L. lactis* spheroplasts. Similarly, the proteins were also incubated with intact *L. lactis* cells.

To study the binding at different pH conditions to pseudomurein of methanogenic archaeal (*Methanobacterium* sp.) cells, 3P-GFP-His_10_ was incubated, washed and resuspended in 50 mM NaH_2_PO_4_, pH 4.0, 6.5 and 9.0. After the incubation and washing steps, the material was placed on a microscope slide and inspected using a phase-contrast microscope (Zeiss Axiophot, Thornwood, USA) fitted with a digital camera and a green filter to view fluorescence. All photographs were taken at 1250-fold magnification.

## Results and Discussion

The nucleotide sequence encoding the PMB domain of the *M. thermautotrophicus* MTH719 protein (amino acid residues 432 to 574; see Fig. 1a) was amplified by PCR and cloned into pBADcLIC [1] using *Escherichia coli* Rosetta gami 2. Fusion genes specifying proteins carrying GFP-His_10_ at the C-terminus of one (1P), two (2P) or three (3P) of the motifs of the PMB domain were made in pBADcLIC-GFP [1] (see Fig. 1b). In-gel fluorescence and Western hybridization of 3P-GFP-His_10_, 2P-GFP-His_10_ and 1P-GFP-His_10_ confirmed expression of the protein in *E. coli* (Figs. S2a, b). Expression of the 3P-His_10_ protein, a fusion of the entire PMB domain to a 10-Histidine tag, was confirmed by SDS-15% PAGE (Fig. S3a) and Western hybridization using anti-His antibodies (Fig. S3b).

### The PMB domain is homogeneous and stable

**Figure 2:**
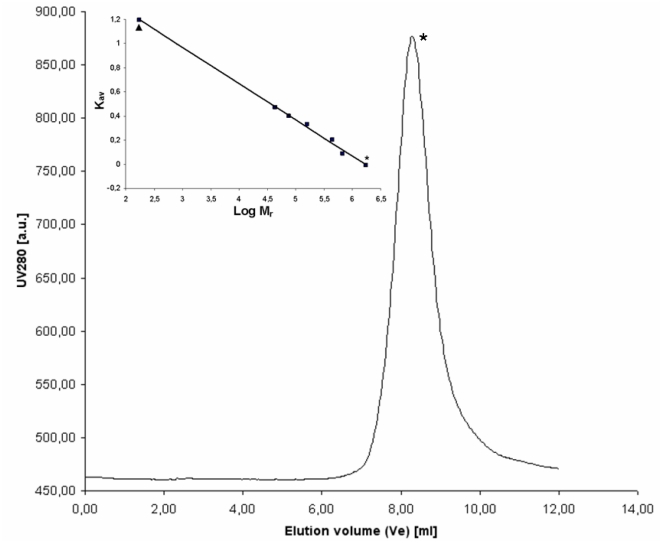
Analytical size exclusion chromatography analysis of the PMB domain 3P-His_10_. Elution profile using a Superdex 200 10/300 GL size exclusion column of Ni-NTA purified 3P-His_10_, eluted with 50 mM NaH_2_PO_4_, 150 mM NaCl, pH 7.0. Inset shows the calibration plot obtained by column calibration with standard globular proteins ranging in size from 43 kDa to 669 kDa. (*) and (^▴^) indicates 3P-His_10_ at pH 7.0 and at pH 9.0 respectively.

**Figure 3:**
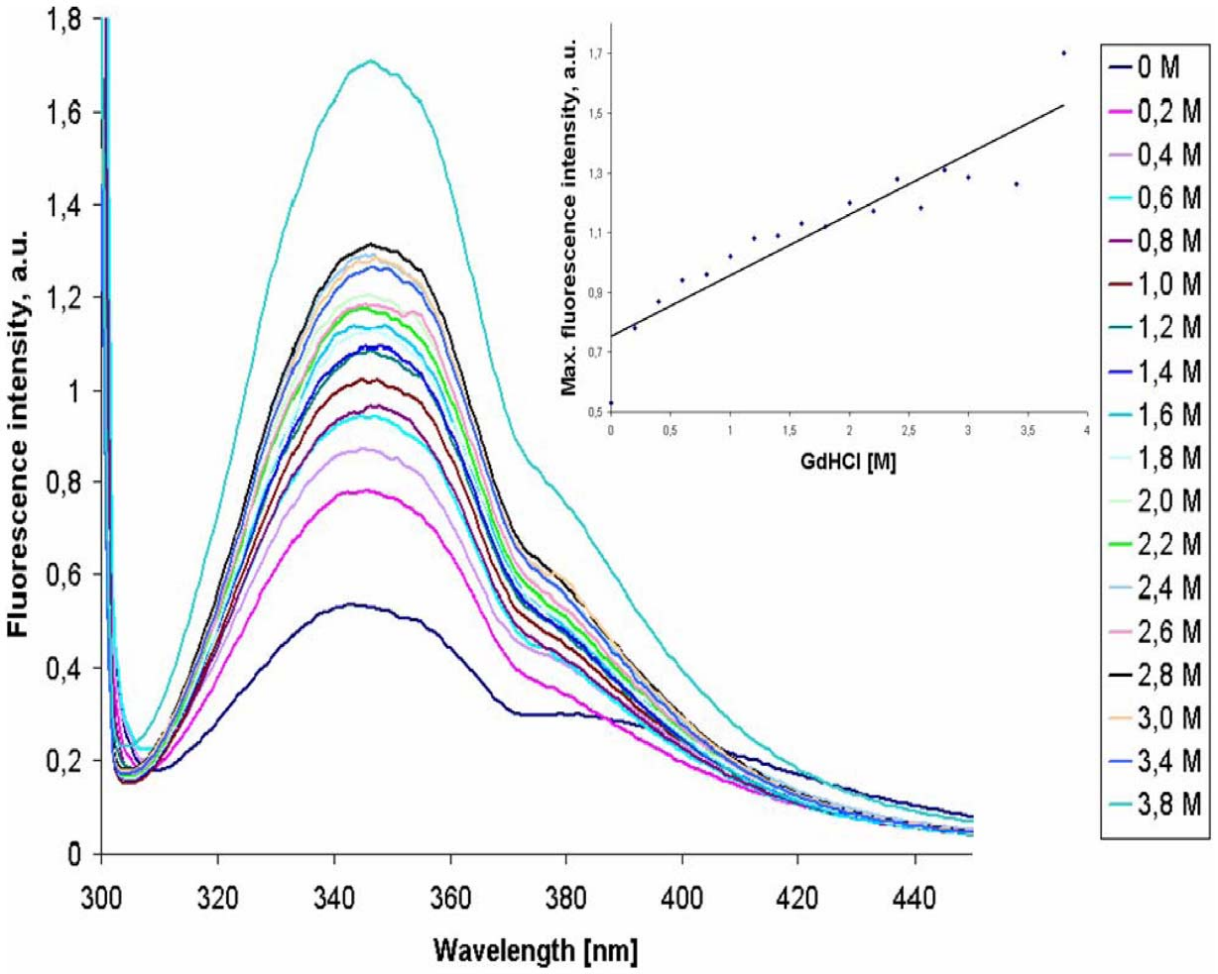
Chemical denaturation of the 3P-His_10_ PMB domain. Tryptophan fluorescence of guanidine hydrochloride (GdHCl)-treated 3P-His_10_ (3 µM in 50 mM NaH_2_PO_4_, pH 8.5) at 25°C. The relative increase of the fluorescence intensity (in arbitrary units, a.u) is shown as a function of the concentration of GdHCl, as indicated in the inset.

3P-His_10_ was purified to homogeneity by Ni-NTA affinity chromatography (Fig. S3c). Native-15% PAGE with different concentrations of purified 3P-His_10_ showed that the protein preparation was homogeneous (data not shown). The protein aggregates into complexes of high molecular mass, as confirmed by analytical size-exclusion chromatography performed at pH 7.0 with known globular protein markers (Fig. 2) Aggregation was not observed at pH 9.0 and the apparent molecular mass of 3P-His_10_ was shown to be 17.4 kDa, which is in agreement with the theoretical molecular mass (18.1 kDa) (Fig. 2 and data not shown). Two tryptophan residues are present in 3P-His_10_, at positions 447 and 507 in the entire protein MTH719. These were employed to study the chemical stability of the domain by tryptophan fluorescence. Chemical denaturation of 3P-His_10_ with guanidine hydrochloride (GdHCl) resulted in maximum fluorescence intensity at 3.8 M GdHCl. This result a gradual increase of tryptophan fluorescence intensity with increasing GdHCl concentration reveals that the PMB motifs fold into a stable domain (Fig. 3). A fluorescence shift of 6 nm (from 342 to 347 nm) was observed when 3P-His_10_ moved from the native to the completely unfolded state (Fig. 3). A small shoulder to the peak that was seen above 370 nm in all the measurements is probably due to the influence of the fourteen tyrosine residues present in the protein domain.

### Proteolytic accessibility of the PMB domain

**Figure 4:**
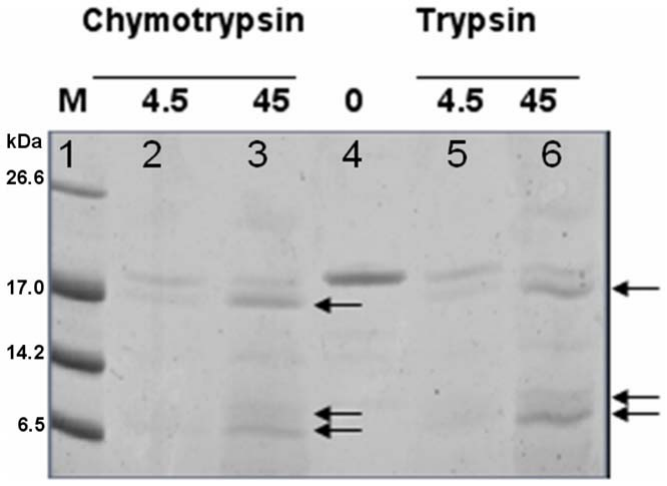
Partial proteolysis of the purified 3P-His_10_, PMB domain. Coomassie brilliant blue-stained SDS-20% PAA gel containing samples of 3P-His_10_ PMB in 50 mM NaH_2_PO_4_, pH 8.5, digested with 4.5 and 45 µM chymotrypsin and trypsin, respectively. Arrows indicate the major digestion products. Lane 1, low range molecular mass marker; lanes 2, 3 and 5, 6 contain chymotrypsin- and trypsin-liberated fragments, respectively; lane 4 contains undigested 3P-His_10_ protein.

Treatment of purified 3P-His_10_ with the proteases chymotrypsin or trypsin resulted in each case in three major protein degradation bands on a SDS-20% PAGE gel and detected by Western blotting using anti-His antibodies (Fig. 4 and data not shown). The observed sizes of the degradation products are in agreement with the prediction of the peptide cutter tool of ExPASy (http://www.expasy.org/tools/), which shows that both enzymes cut once between motifs one and two. Additionally, chymotrypsin cleaves at the Tev protease cleavage site immediately upstream of the His_10_ tag while trypsin cleaves close to the N-terminus of 3P-His_10_, removing the short “MGGGFA” peptide region resulting from the cloning procedure [1]. Both of these small fragments would not be visible on the SDS-20% PAGE gel. Although there are a number of other predicted cleavage sites in the motif regions of the PMB domain cleavage at these positions is not observed. Apparently, the motif region of the PMB domain is protected from these proteases. These results support the previous result that the PMB motifs fold into a stable domain, thus protecting the other possible cleavage sites.

### The PMB domain binds to pseudomurein of methanogenic archaeal cells as well as to bacterial spheroplasts

**Figure 5:**
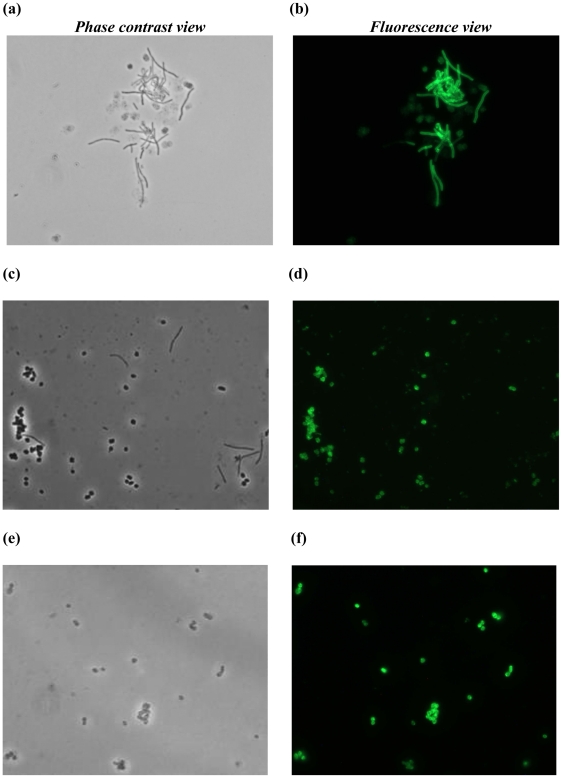
PMB domain binding to pseudomurein of methanogenic archaeal cells and bacterial spheroplasts. (**a**) Phase contrast and (**b**) fluorescence microscopy view of 3P-GFP-His_10_ bound to pseudomurein of *Methanobacterium* sp. (long rods can still be seen in the cell extract) and *L. lactis* spheroplasts (ovoid). (**c**) Phase contrast and (**d**) fluorescence microscopy view of 2P-GFP-His_10_, bound only to *L. lactis* spheroplasts, in a mixture with pseudomurein of *Methanobacterium* sp. cells (long rods). (**e**) Phase contrast and (**f**) fluorescence microscopy view of the PMB domain of PeiW fused with GFP-His_6_ bound to *L. lactis* spheroplasts.

The Ni-NTA purified protein 3P-GFP-His_10_ was incubated with pseudomurein-containing archaeal cells (*Methanobacterium* sp.) and purified murein *i.e*., *L. lactis* GEM (Gram-positive Enhancer Matrix) particles [6] at room temperature. The fusion protein bound to the surface of these archaeal cells (Fig. 5a, b) but not to GEM particles (data not shown). Previously, it was shown that the PMB domain of PeiW from the archaeal endoisopeptidase bind to pseudomurein-containing whole archaeal cells but not to whole cells of bacteria [2]. This affinity of the PMB domain for methanogenic archaeal cells is probably due to the presence of pseudomurein in the archaeal cell envelope, which is lacking in bacteria [2], [7], [12]. Surprisingly, when we added the 3P-GFP-His_10_ and the PMB domain of PeiW fused to GFP-His_6_ proteins to lysozyme-treated *L. lactis* cells (spheroplasts), binding was observed to these spheroplasts (Fig. 5a, b, e & f). The fluorescent signal could not be removed from the cell fragments with 150 mM NaCl, indicating that the binding is firm and specific. Binding was also observed to spheroplasts of the Gram-negative bacterium *E. coli* (data not shown). Since lysozyme treatment of murein generates fragments with free NAM and/or NAG residues and NAG is the only common moiety between murein and pseudomurein, we presume that the PMB domain, like the LysM domain [3], [4], recognizes the NAG moiety. The reason why the PMB domain does not recognize the intact bacterial cell wall is not known. It could be that the composition (NAM and NAT) and the bonding of these two sugars with NAG [β(1–4) and β(1–3)] in murein and pseudomurein, respectively, are different and that these small changes influence the chemistry of the final product. Unfortunately three-dimensional structures are not yet available for the PMB domain or for PMB-containing proteins, which could help to explain these observations.

### The PMB domain requires at least three motifs for binding to pseudomurein of methanogenic archaeal cells and the binding is pH dependent

**Figure 6:**
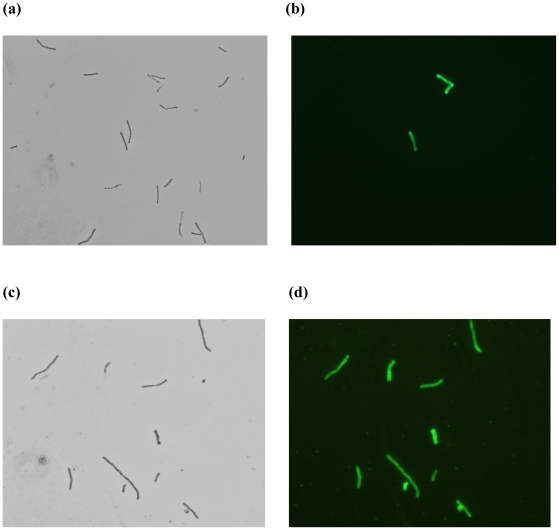
Binding of 3P-GFP-His_10_ to pseudomurein of methanogenic archaeal cells at different pH conditions. (**a**) Phase contrast and (**b**) fluorescence microscopy views of 3P-GFP-His_10_ bound to pseudomurein of *Methanobacterium* sp. cells (long rods) at pH 6.5. (**c**) Phase contrast and (**d**) fluorescence microscopy views of 3P-GFP-His_10_ bound to pseudomurein of *Methanobacterium* sp. cells at pH 9.0.

The purified proteins 3P-GFP-His_10_, 2P-GFP-His_10_ and 1P-GFP-His_10_ were tested for their ability to bind to the methanogenic archaeal cell envelope (pseudomurein of *Methanobacterium* sp. cells), to bacterial cell envelopes (whole cells of *L. lactis* and *E. coli*) as well as to spheroplasts of *L. lactis*. NaCl (150 mM) was added in all the reactions in order to remove any unspecific binding. Although 3P-GFP-His_10_ was able to bind to both pseudomurein-containing archaeal cells and to *L. lactis* spheroplasts (Fig. 1b and 5a, b), 2P-GFP-His_10_ bound to the later only, not to pseudomurein of methanogenic archaeal cells (Fig. 1b and 5c, d). 1P-GFP-His_10_ failed to bind to either pseudomurein-containing archaeal cells or to the *L*. *lactis* spheroplasts (Fig. 1b and data not shown). Apparently, the PMB domain can bind to methanogenic archaea only when it contains three motifs. The fusion proteins with 2 or 3 of the PMB motifs were able to bind to *L. lactis* spheroplasts, while none of the fusion proteins bound to intact bacterial cells (Fig. 1b). Fully functional, purified 3P-GFP-His_10_ was tested for binding to pseudomurein of methanogenic archaeal cells (*Methanobacterium* sp.) at pH 4.0, 6.5 and 9.0. At these 3 pH's the GFP signal was not affected for a number of GFP-fusion proteins tested (data not shown). No binding was observed at pH 4.0 (data not shown). At pH 6.5, only partial binding was seen, as either only a few archaeal cells were fluorescent (Fig. 6a, b) or a low GFP signal was observed (data not shown). At pH 9.0, which is close to the pI value of the 3P-GFP-His_10_ protein (pI 9.2), complete binding was observed (Fig. 6c, d). The decreased binding seen at pH 6.5 might be due a lower effective concentration of 3P-GFP-His_10_ due to the aggregation of the protein at low pH conditions (pH 7.0) (Fig. 2). GFP protein (control), purified by hydrophobic interaction chromatography (HIC), did not bind to pseudomurein of methanogenic archaeal cells nor to *L. lactis* spheroplasts at all 3 pH's (data not shown), indicating that the PMB domain is functionally active at a pH close to its pI and that binding is strongly influenced by the pH of the binding environment.

Summarizing, we conclude that the pseudomurein cell wall-binding domain performs a function similar to that of the LysM domain. The three C-terminal PMB motifs of MTH719 fold into a stable pseudomurein cell wall-binding domain which binds not only to pseudomurein of methanogenic archaeal cells (requiring at least three motifs) but also to the cell wall fragments on bacterial spheroplasts, for which two motifs are enough.

## Supporting Information

Figure S1 **Rapid spheroplast lysis test.** Phase contrast microscopic views of *Lactococcus lactis* cells untreated (**a**) and treated (**b**) with lysozyme. Spheroplasts instantaneously lysed upon addition of 0.5% SDS (c) while the untreated cells did not, even after addition of 2.5% SDS (data not shown).
(DOC)

Figure S2 **Isolation of MTH719 PMB-GFP fusion variants.** (**a**) Western blot generated using anti-His-antibodies. Ni-NTA-purified 3P-GFP-His_10_ (47.2 kDa), 2P-GFP-His_10_ (43.2 kDa) and 1P-GFP-His_10_ (35.2 kDa) proteins were applied in lanes 1–3, respectively; Lane 4, PeiW-His_6_ (35.4 kDa) control. Block arrows indicate the specific protein band. The extra bands in lanes 1, 2 and 4 are most likely break down products that are devoid of functional GFP and do not fluoresce (Fig. S2b). (**b**) In-gel fluorescence. Lanes (1–3), in-gel fluorescence of MTH719 (3P-GFP-His_10_, 2P-GFP-His_10_ and 1P-GFP-His_10_) respectively, lane 4, PeiW-GFP (60.5 kDa) positive control, (**→**) indicates the specific fluorescent protein band.
(DOC)

Figure S3 **Expression of the 3P-His_10_ PMB domain.** (**a**) SDS-15% PAGE; lane 1, Low molecular mass marker; lanes 2 and 3, cell-free extract from *E. coli* Rosetta gami 2 cells without plasmid, uninduced and induced with 0.2% arabinose, respectively; lanes 4 and 5, cell-free extracts from *E. coli* Rosetta gami 2 carrying a plasmid specifying the 3P-His_10_ PMB domain, uninduced and induced with 0.2% arabinose, respectively. The block arrow indicates the proper protein band. (**b**) Western blot decorated with anti-His antibodies. Lanes 1 and 2, uninduced and 0.2% arabinose-induced 3P-His_10_ PMB domain (18.7 kDa), respectively; lane 3, SpoOA-His_6_ (15.2 kDa): His-tagged positive control protein purified from *Bacillus subtilis* [13]. (**c**) SDS-15% PAGE showing the Ni-NTA elution profile of the 3P-His_10_ PMB domain; lane 1, Low molecular mass marker; lanes 2 to 7 are the six elution fractions.
(DOC)

Table S1 **Primers for the construction of MTH719 PMB gene fusions used in this study.** Bases denoted in italics were added before the gene sequence to each forward and reverse primer as described by [1]. The bases in bold are the starting and reverse complementary bases for 3P-His_10_, 3P-GFP-His_10_, 2P-GFP-His_10_ and 1P-GFP-His_10_. 3PF/2PF was the forward primer for 3P-His_10_, 3P-GFP-His_10_ and 2P-GFP-His_10_ constructs. 3PR/1PR was the reverse primer for 3P-His_10_, 3P-GFP-His_10_ and 1P-GFP-His_10_ constructs. 2PR and 1PF were the reverse and forward primers of 2P-GFP-His_10_ and 1P-GFP-His_10_, respectively.
(DOC)
