# Supplementary figures and images for: A Minimum of Three Motifs Is Essential for Optimal Binding of Pseudomurein Cell Wall-Binding Domain of Methanothermobacter thermautotrophicus
Source: PLoS One. 2011 Jun 27;6(6):e21582. doi: 10.1371/journal.pone.0021582 (PMC3124540; doi:10.1371/journal.pone.0021582)

**Figure S1.**

**(a)**

**
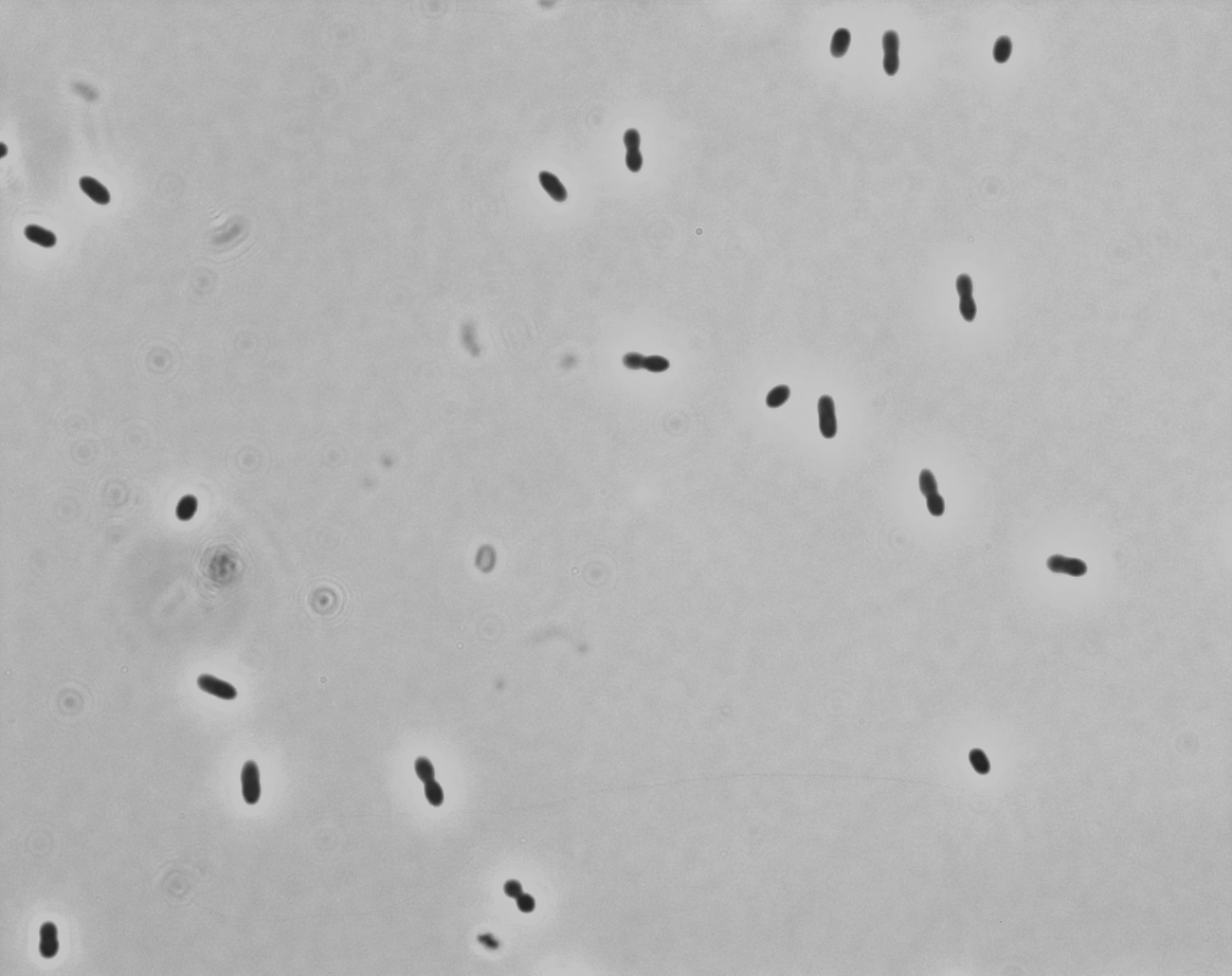
**

**(b)**

**
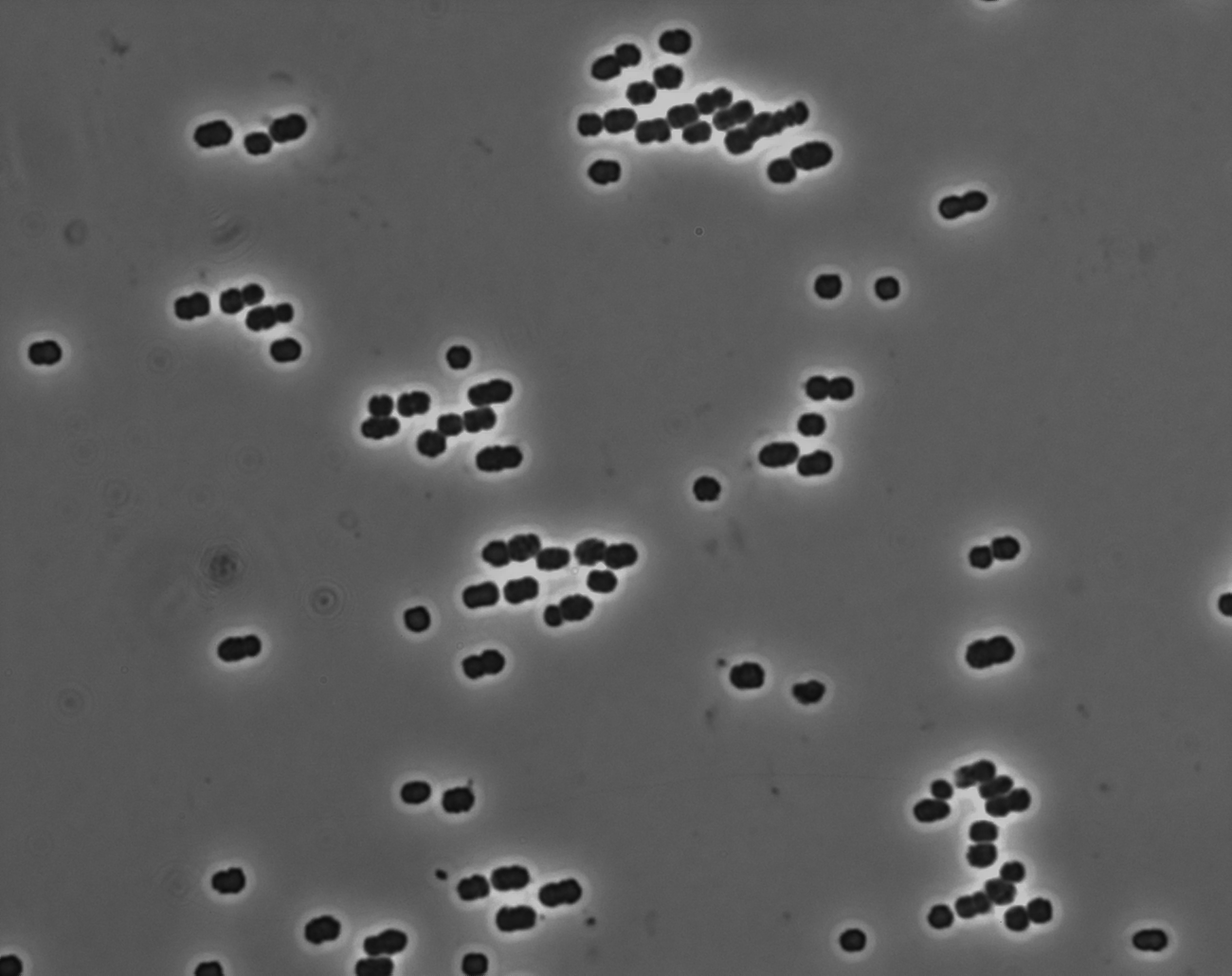
**

**(c)**

**
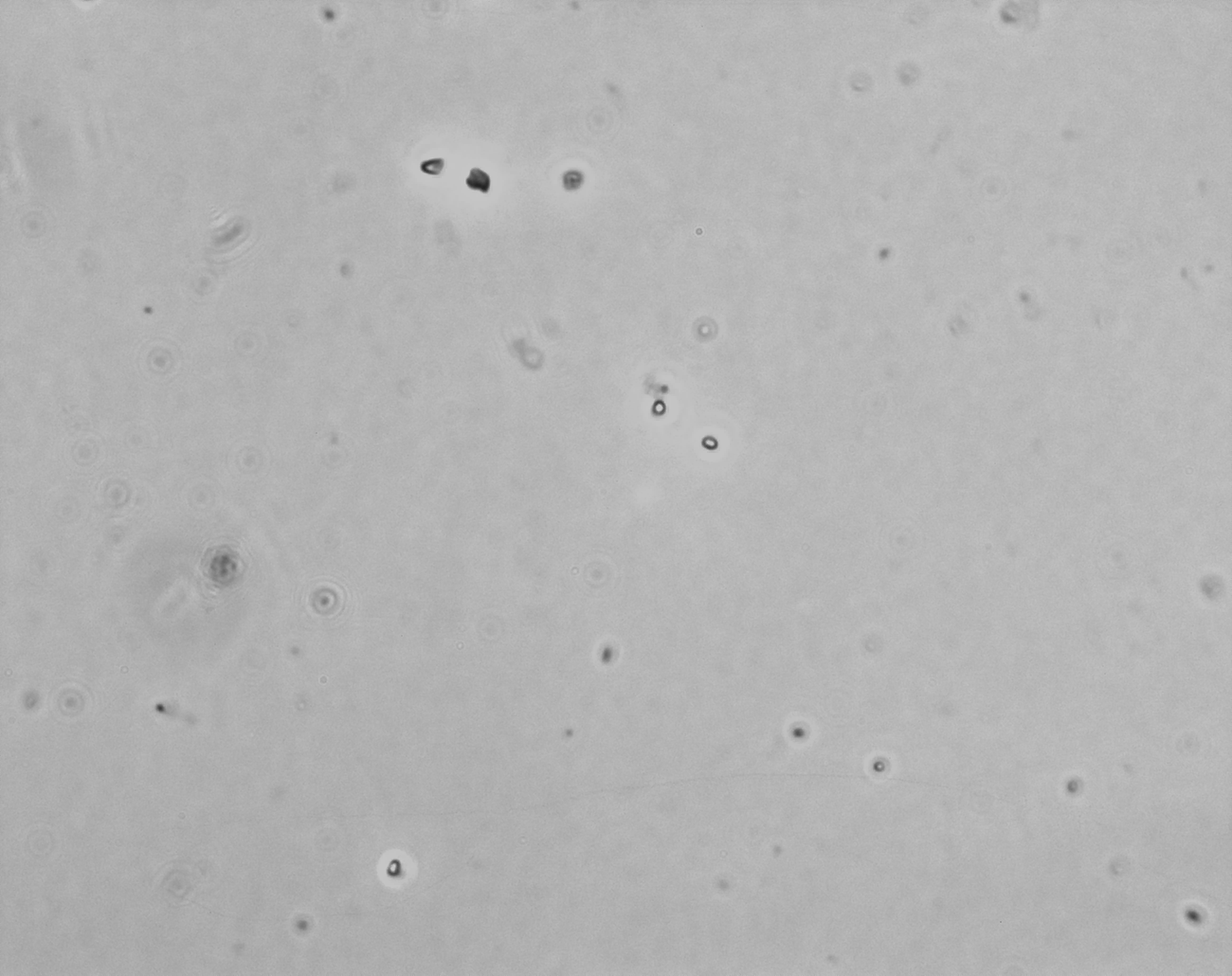
**

Supplement: Figure S1 — Rapid spheroplast lysis test. Phase contrast microscopic views of Lactococcus lactis cells untreated (a) and treated (b) with lysozyme. Spheroplasts instantaneously lysed upon addition of 0.5% SDS (c) while the untreated cells did not, even after addition of 2.5% SDS (data not shown). (DOC) [file pone.0021582.s001.doc]

**Figure S2.**

**(a)**


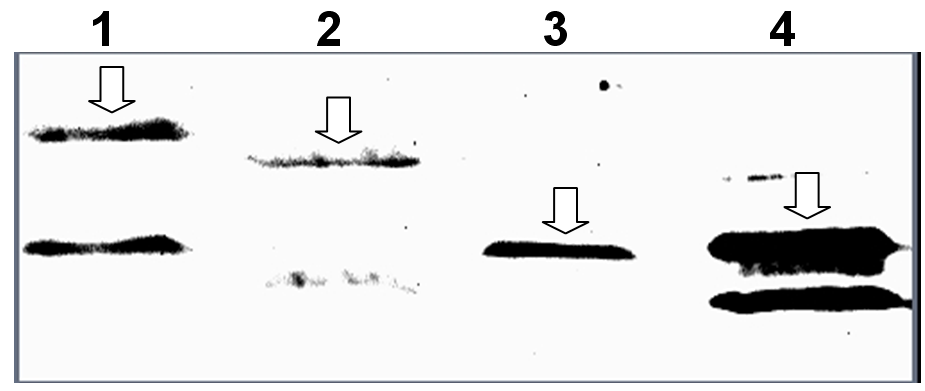


**(b)**

**
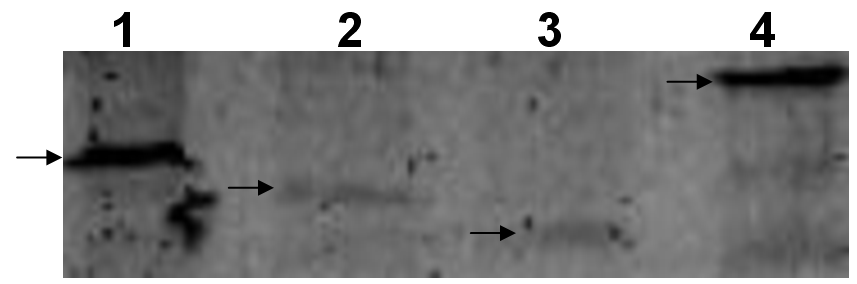
**

Supplement: Figure S2 — Isolation of MTH719 PMB-GFP fusion variants. (a) Western blot generated using anti-His-antibodies. Ni-NTA-purified 3P-GFP-His10 (47.2 kDa), 2P-GFP-His10 (43.2 kDa) and 1P-GFP-His10 (35.2 kDa) proteins were applied in lanes 1–3, respectively; Lane 4, PeiW-His6 (35.4 kDa) control. Block arrows indicate the specific protein band. The extra bands in lanes 1, 2 and 4 are most likely break down products that are devoid of functional GFP and do not fluoresce (Fig. S2b). (b) In-gel fluorescence. Lanes (1–3), in-gel fluorescence of MTH719 (3P-GFP-His10, 2P-GFP-His10 and 1P-GFP-His10) respectively, lane 4, PeiW-GFP (60.5 kDa) positive control, (→) indicates the specific fluorescent protein band. (DOC) [file pone.0021582.s002.doc]

**Figure S3.**

**(a)**

**
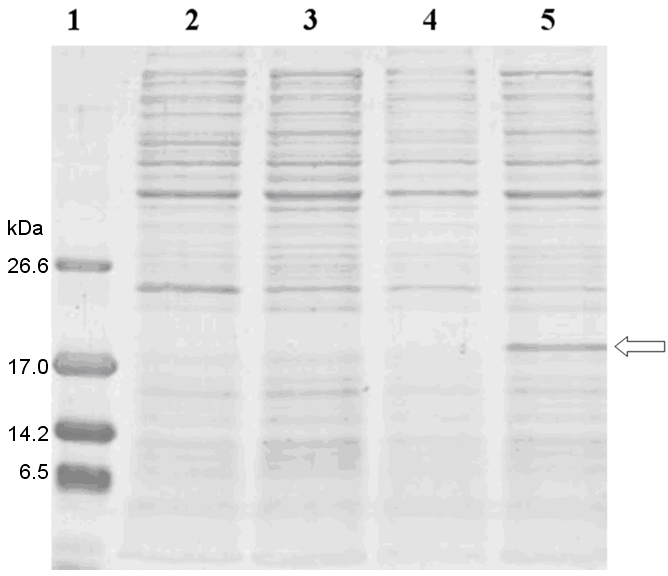
**

**(b)**


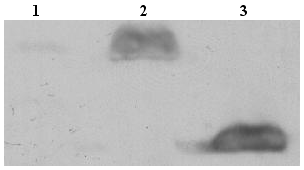


**(c)**

**
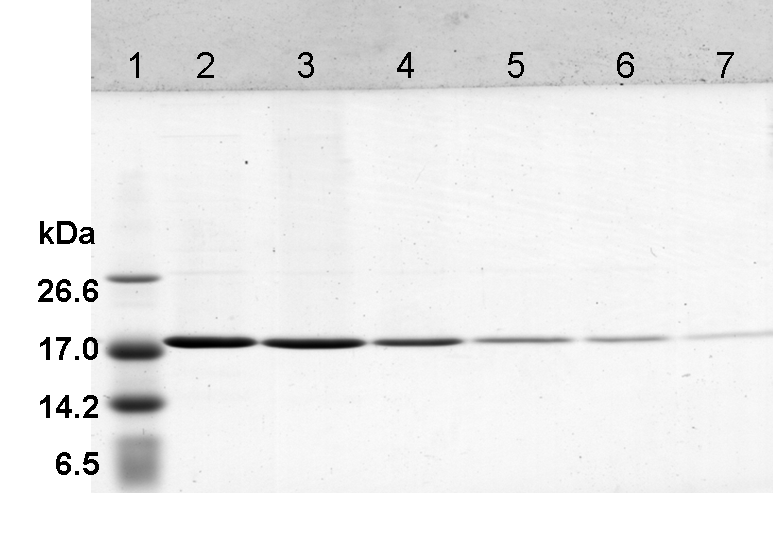
**

Supplement: Figure S3 — Expression of the 3P-His10 PMB domain. (a) SDS-15% PAGE; lane 1, Low molecular mass marker; lanes 2 and 3, cell-free extract from E. coli Rosetta gami 2 cells without plasmid, uninduced and induced with 0.2% arabinose, respectively; lanes 4 and 5, cell-free extracts from E. coli Rosetta gami 2 carrying a plasmid specifying the 3P-His10 PMB domain, uninduced and induced with 0.2% arabinose, respectively. The block arrow indicates the proper protein band. (b) Western blot decorated with anti-His antibodies. Lanes 1 and 2, uninduced and 0.2% arabinose-induced 3P-His10 PMB domain (18.7 kDa), respectively; lane 3, SpoOA-His6 (15.2 kDa): His-tagged positive control protein purified from Bacillus subtilis [13]. (c) SDS-15% PAGE showing the Ni-NTA elution profile of the 3P-His10 PMB domain; lane 1, Low molecular mass marker; lanes 2 to 7 are the six elution fractions. (DOC) [file pone.0021582.s003.doc]
