# Supplementary material for: A Minimum of Three Motifs Is Essential for Optimal Binding of Pseudomurein Cell Wall-Binding Domain of Methanothermobacter thermautotrophicus
Source: PLoS One. 2011 Jun 27;6(6):e21582. doi: 10.1371/journal.pone.0021582 (PMC3124540; doi:10.1371/journal.pone.0021582)
Supplement: Table S1 — Primers for the construction of MTH719 PMB gene fusions used in this study. Bases denoted in italics were added before the gene sequence to each forward and reverse primer as described by [1]. The bases in bold are the starting and reverse complementary bases for 3P-His10, 3P-GFP-His10, 2P-GFP-His10 and 1P-GFP-His10. 3PF/2PF was the forward primer for 3P-His10, 3P-GFP-His10 and 2P-GFP-His10 constructs. 3PR/1PR was the reverse primer for 3P-His10, 3P-GFP-His10 and 1P-GFP-His10 constructs. 2PR and 1PF were the reverse and forward primers of 2P-GFP-His10 and 1P-GFP-His10, respectively. (DOC) [file pone.0021582.s004.doc]

**Table S1. Primers used for the construction of MTH719 PMB gene fusions used in this study.**

| **Oligo** | **Primer sequence** |
| --- | --- |
| 3PF/2PF | *ATGGGTGGTGGATTTGCT***GCGAGTGTTAAAATTAGTG** |
| 3PR/1PR | *TTGGAAGTATAAATTTTC***GATTGTGACGTAGTTTGGC** |
| 2PR | *TTGGAAGTATAAATTTTC***GATGGCGTAATTTGGTGC** |
| 1PF | *ATGGGTGGTGGATTTGCT***GGCCTTGGAAGAATACCC** |
